# Ebola in West Africa. Before, now and then

**DOI:** 10.11694/pamj.supp.2015.22.1.8145

**Published:** 2015-10-10

**Authors:** Raoul Kamadjeu

**Affiliations:** 1 The Pan African Medical Journal, Nairobi, Kenya

**Keywords:** Ebola, EVD, West Africa, Health system, outbreak

## Editorial

In December 2013, a case of Ebola was reported from Guéckédou, a forested area of Guinea near the border with Liberia and Sierra Leone. In 2014, the worst epidemic of Ebola Virus Disease (EVD) the world has ever seen is still ongoing in West Africa. According to the World Health Organization Ebola situation report [1], more than 28,400 cases and 11,300 fatalities have been reported from 6 countries in West Africa by September 2015.

The disease laid bare the extreme weakness of the health care system in these countries; consequence of decades of civil war, chronic underfunding, mismanagement and chronic underdevelopment. The trail of devastation left behind by the disease will be felt for the years to come: the socio-economic fabric of the affected countries was shattered putting development targets in jeopardy; time honored traditions and ways of life were revoked, community trust in health care service was damaged and the meager healthcare workforce at the forefront in the fight against the outbreak was decimated. From an initial slow and highly criticized global response; the EVD outbreak became a critical turning point in global outbreak response; it tested the ability of various organizations, charities and national governments to collaborate in an unprecedented way. The Ebola outbreak was a wakeup call for national governments and the international community to strengthen preparedness and response capacities in Africa and elsewhere in order to deal with the inevitable next outbreak.

A lot has been said on the lessons African governments and the international community should draw from the Ebola outbreak. Topping that list is the need to build or rebuild strong and resilient health care systems backed by a well-trained and highly motivated workforce and able to provide basic health services to and with the communities and fit to timely detect and respond to diseases of epidemic potential. The outbreak also highlighted the need to accelerate research and development around the diagnostic, treatment and vaccination against EVD. While resilient population in Liberia, Sierra Leone and Guinea are picking up the pieces and going through what will be a long the recovery process, African governments should lean towards a continentally or regionally focused approach to public health response; this can be done through existing continental and regional bodies or mechanisms, with support of traditional and new partners. Another outbreak will hit, but when it does, it should not be business as usual, it should find us prepared.
